# Effects of Vaccine Program against Pandemic Influenza A(H1N1) Virus, United States, 2009–2010

**DOI:** 10.3201/eid1903.120394

**Published:** 2013-03

**Authors:** Rebekah H. Borse, Sundar S. Shrestha, Anthony E. Fiore, Charisma Y. Atkins, James A. Singleton, Carolyn Furlow, Martin I. Meltzer

**Affiliations:** Author affiliation: Centers for Disease Control and Prevention, Atlanta, Georgia, USA

**Keywords:** Influenza, viruses, vaccine, vaccination, A(H1N1)pdm09, H1N1, pandemic, model

## Abstract

In April 2009, the United States began a response to the emergence of a pandemic influenza virus strain: A(H1N1)pdm09. Vaccination began in October 2009. By using US surveillance data (April 12, 2009–April 10, 2010) and vaccine coverage estimates (October 3, 2009–April 18, 2010), we estimated that the A(H1N1)pdm09 virus vaccination program prevented 700,000–1,500,000 clinical cases, 4,000–10,000 hospitalizations, and 200–500 deaths. We found that the national health effects were greatly influenced by the timing of vaccine administration and the effectiveness of the vaccine. We estimated that recommendations for priority vaccination of targeted priority groups were not inferior to other vaccination prioritization strategies. These results emphasize the need for relevant surveillance data to facilitate a rapid evaluation of vaccine recommendations and effects.

## Abstract 2

Vaccination likely prevented 700,000–1,500,000 clinical cases, 4,000–10,000 hospitalizations, and 200–500 deaths.

On April 26, 2009, the United States declared a public health emergency in response to the 2009 pandemic influenza A(H1N1)pdm09 virus (1). The Centers for Disease Control and Prevention (CDC) estimated that in the United States during April 12, 2009–April 10, 2010, there were 61 million clinical cases of influenza and that 274,000 persons were hospitalized and 12,500 died (2). For the purpose of this study, we considered clinical cases as influenza-like illness in persons who did or did not seek medical care (2).

The US Food and Drug Administration approved multiple formulations of monovalent inactivated, unadjuvanted influenza vaccine, and a monovalent live attenuated vaccine against A(H1N1)pdm09 virus in mid-September 2009 (3); a national vaccination program was initiated in October (4). In July 2009, estimating that initial vaccine supplies could be insufficient to meet demand, the Advisory Committee on Immunization Practices (ACIP) recommended priority groups for the vaccination program. These priority groups included pregnant women, household contacts and caregivers of children <6 months of age, health care and emergency medical services personnel, all persons 6 months–24 years of age, persons <19 years of age who were receiving long-term aspirin therapy, and persons 25–64 years of age who had health conditions associated with a higher risk for medical complications from influenza. Such complications include asthma; neurodevelopmental conditions; chronic lung disease; heart disease; blood, endocrine, kidney, liver, and metabolic disorders, and a weakened immune system. (5,6). When a vaccine against the pandemic strain was released for initial use, the supply was only 25%–50% of the amount that had been projected because vaccine production yields were lower than expected (7,8). By January 2010, when 100 million doses had been delivered and an estimated 57 million doses had been administered (9), most states were offering vaccination to anyone >6 months of age. By February 2010, 125 million doses, most of which were inactivated, had been made available and ≈69 million persons had been vaccinated (4,9,10). Final estimates indicated that by the end of May 2010, ≈81 million persons had been vaccinated and 90 million doses had been administered (11).

We estimated the number of clinical cases, hospitalizations, and deaths prevented in the United States that were directly attributable to the 2009–2010 A(H1N1)pdm09 virus vaccination program. These results can be used by public health officials, policy makers, and the public to evaluate this program and plan for the management of future pandemics.

## Methods

### Calculation Overview

Using Excel (Microsoft Corp., Redmond, Washington, USA), we developed a tool to estimate the effects of the vaccination program (Technical Appendix). The estimate was based on the actual epidemic curve in the United States, which included the effects of the vaccination program. We divided the US population into 8 subgroups: 1) persons 6 months–9 years of age; 2) persons 10–24 years (all persons 10–17 years of age and persons 18–24 years, not pregnant); 3) pregnant women, 18–64 years; 4) persons 25–64 years, high risk, not pregnant; 5) persons 25–64 years, health care workers, non–high risk, not pregnant; 6) persons 25–64 years who had contact with a child <6 months of age, non–health care worker, non–high risk, not pregnant; 7) persons 25–64 years who did not have contact with a child <6 months of age, non-health care worker, non-high risk, not pregnant; and 8) persons >65 years.

**Figure 1:**
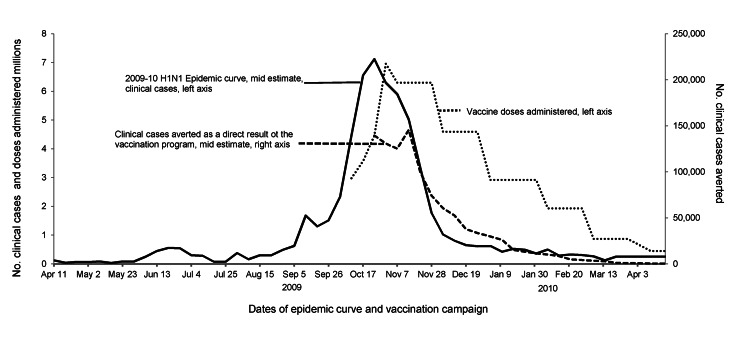
Weekly number of clinical cases of influenza A(H1N1)pdm09 virus infection, the number of vaccine doses administered, and the estimated number of cases averted over time because of the vaccination program. Midranges shown for epidemic curve and clinical cases; ranges provided in Table 3.

First, we calculated the weekly number of vaccine doses administered within each population subgroup. We then estimated, using the existing epidemic curve, the probability that a person who was vaccinated had not previously been infected with A(H1N1)pdm09 virus and had a clinical or subclinical case and the probability that a person would be infected during the remaining portion of the season. We adjusted our estimates for a 2-week delay in protection against the virus after vaccine administration (12). In this initial calculation (phase 1), we based the probability of infection on the actual epidemic curves during the pandemic, April 11, 2009–April 18, 2010 (13) (Figure 1), because those were the best sources of data available. This calculation included the effects of the vaccination program, as described below in Equations 1a and 1b.

During phase 2 of the calculation, we adjusted the probabilities of infection over time to include the number of averted events by including the event prevented (i.e., clinical cases, hospitalizations, or deaths) in the epidemic curve (see Equations 2a, 2b below). Our original epidemic curve included the effects of the actual vaccination program; therefore, it was necessary to repeat the second phase (i.e., continue to add the number of clinical events into the epidemic curve) until the number of events in the final epidemic curve (final estimate from Equation 2b), minus the number of events prevented (final estimate from Equation 2a), exactly matched the epidemic curve that existed during the pandemic, week by week, for each population subgroup. This enabled us to estimate the direct effects of the vaccination program.

### Data

#### Demographics

The population in each ACIP-defined prioritized target group was estimated by using the National 2009 H1N1 Flu Survey (NHFS) (14–16) and CDC’s 2008–09 projected influenza vaccination target population sizes (17) (Table 1). The total population of pregnant women over the course of the pandemic was based on data from Moro et al. (18).

**Table 1:** Data used to calculate effects of vaccination program against influenza A(H1N1)pdm09 virus by population subgroup*

Subgroup	Population	% Vaccinated	No. doses recommended for full coverage	% Assumed outcomes		
				Vaccine effectiveness†	Clinical cases and hospitalizations	Deaths
6 mo–9 y	39,429,115	1st dose/45, 2nd dose/23	2 doses, 4 wks apart	1st dose/0, 2nd dose/62	20.1	6.0
10–24 y (10–17 all, 18–24 NP)	59,684,833	27	1	62	22.3	8.0
Pregnant 18–64 y	5,578,782	43	1	62	2.2	4.0
25–64 y, HR, NP	33,949,395	27	1	62	13.5	24.0
25–64 y, HCW, non-HR, NP	17,451,921	36	1	62	5.6	9.0
25–64 y, contact <6 mo, non-HCW, non-HR, NP	8,933,718	23	1	62	2.5	6.0
25–64 y, non-contact <6 mo, non-HCW, non-HR, NP	96,235,755	16	1	62	24.0	30.0
>65 y	37,989,965	28	1	43	9.8	13.0
Total	299,253,484	27	1–2	NA	NA	NA
References	(14,28,39)	(9,14,28)	(6)	(24,36,40)	(2,19–21)	(2,19–21)

*NP, not pregnant; HR, high risk; HCW, health care worker; contact, household contacts and caregivers of children <6 months of age; NA, not applicable. †Data are for effectiveness against clinical cases, hospitalizations, and deaths. For population subgroup 6 mo–9 y, we assumed the vaccine reached effectiveness levels 2 wk after full coverage (12).

#### Clinical Cases, Hospitalizations, and Deaths

Three influenza surveillance systems in the United States were used to estimate the incidence and outcomes of A(H1N1)pdm09; the detailed methods are published in Shrestha et al. (2). The ranges of our data are based on the ranges of these epidemic curves (Figure 1). We reviewed published estimates and expert opinion (2, 19–21) (Table 1) to estimate the proportion (Table 1) and thus the incidence over time (used in Equation 1a) of A(H1N1)pdm09-related clinical cases, hospitalizations, and deaths for each population subgroup after being vaccinated,.

#### Vaccine-related

Our estimates of vaccination coverage were based on combined monthly data from the NHFS and the Behavioral Risk Factor Surveillance System survey (9,22). Children <10 years of age required 2 doses; we assumed that children who received their second dose received it 4 weeks after their first dose (23) (Table 1).

Our estimates of vaccine effectiveness are based on studies from Europe and China (24–27) and expert opinion based on unpublished internal CDC studies (Table 1). On the basis of these data, we assumed that the vaccine was 62% effective in protecting against clinical cases, hospitalizations, and deaths for all population subgroups except for persons >65, for whom we assumed the vaccine to be 43% effective (Table 1). To date, there are no published data from the United States that reflect calculations of vaccine effectiveness of an unadjuvanted A(H1N1)pdm09 virus vaccine on clinical cases, hospitalizations, or deaths. We further assumed that persons vaccinated were not protected from the A(H1N1)pdm09 virus until 2 weeks after the final dose (1 dose for persons ≥10 years, 2 doses for children <10 years) (12). We estimated the number of persons vaccinated, by population subgroup, based on data reported to CDC in the NHFS and the Behavioral Risk Factor Surveillance System survey October 3, 2009–April 18, 2010 (4,9,10,15,22,28) (Table 2).

**Table 2:** Estimates of cumulative weekly number of influenza A(H1N1)pdm09 virus vaccine doses administered (rounded to the nearest 1,000), by population subgroup*

Week‡	Week ending	No. persons vaccinated	No. doses	Estimated doses by population subgroup†								
				Age 6 mo–9 y		Age 10–24 y	Pregnant, Age 18–64 y§	Age 25–64 y				Age >65 y
				1st dose	2nd dose			HR, NP	HCW, non-HR, NP	Contact with children <6 mo, non-HCW, non-HR, NP	No contact with children <6 mo, non-HCW, non-HR, NP	
40	2009 Oct 10	2,971,000	2,971,000	836,000	0	578,000	171,000	314,000	283,000	69,000	363,000	358,000
41	2009 Oct 17	6,536,000	6,536,000	1,838,000	0	1,272,000	376,000	690,000	622,000	151,000	799,000	788,000
42	2009 Oct 24	10,993,000	10,993,000	3,091,000	0	2,139,000	633,000	1,161,000	1,046K	254K	1,343K	1,325K
43	2009 Oct 31	17,826,000	17,944,000	5,013,000	119,000	3,469,000	1,026,000	1,882,000	1,696,000	412,000	2,178,000	2,148,000
44	2009 Nov 7	23,755,000	24,239,000	6,526,000	483,000	4,776,000	1,230,000	2,467,000	2,275,000	573,000	3,116,000	2,790,000
45	2009 Nov 14	29,684,000	30,533,000	8,040,000	848,000	6,084,000	1,435,000	3,053,000	2,853,000	734,000	4,054,000	3,433,000
46	2009 Nov 21	35,613,000	36,827,000	9,553,000	1,213,000	7,391,000	1,639,000	3,638,000	3,431,000	895,000	4,991,000	4,075,000
47	2009 Nov 28	41,543,000	43,121,000	11,067,000	1,578,000	8,698,000	1,843,000	4,223,000	4,009,000	1,056,000	5,929,000	4,717,000
48	2009 Dec 5	45,427,000	47,709,000	11,837,000	2,282,000	9,511,000	1,911,000	4,717,000	4,275,000	1,146,000	6,799,000	5,231,000
49	2009 Dec 12	49,311,000	52,297,000	12,608,000	2,985,000	10,325,000	1,978,000	5,211,000	4,540,000	1,235,000	7,669,000	5,745,000
50	2009 Dec 19	53,196,000	56,885,000	13,379,000	3,689,000	11,138,000	2,045,000	5,705,000	4,805,000	1,325,000	8,539,000	6,259,000
51	2009 Dec 26	57,080,000	61,473,000	14,150,000	4,393,000	11,952,000	2,112,000	6,199,000	5,071,000	1,414,000	9,409,000	6,773,000
52	2010 Jan 3	59,526,000	64,388,000	14,541,000	4,862,000	12,406,000	2,171,000	6,480,000	5,217,000	1,463,000	10,031,000	7,217,000
1	2010 Jan 10	61,972,000	67,304,000	14,931,000	5,332,000	12,860,000	2,230,000	6,760,000	5,364,000	1,513,000	10,653,000	7,661,000
2	2010 Jan 17	64,417,000	70,219,000	15,322,000	5,802,000	13,313,000	2,289,000	7,040,000	5,510,000	1,562,000	11,275,000	8,105,000
3	2010 Jan 24	66,863,000	73,134,000	15,713,000	6,271,000	13,767,000	2,349,000	7,320,000	5,657,000	1,611,000	11,897,000	8,550,000
4	2010 Jan 31	69,309,000	76,050,000	16,103,000	6,741,000	14,221,000	2,408,000	7,601,000	5,803,000	1,660,000	12,519,000	8,994,000
5	2010 Feb 7	70,924,000	77,979,000	16,353,000	7,055,000	14,489,000	2,408,000	7,846,000	5,863,000	1,702,000	12,999,000	9,263,000
6	2010 Feb 14	72,539,000	79,907,000	16,603,000	7,369,000	14,757,000	2,408,000	8,092,000	5,924,000	1,745,000	13,479,000	9,532,000
7	2010 Feb 21	74,154,000	81,836,000	16,852,000	7,683,000	15,025,000	2,408,000	8,338,000	5,984,000	1,787,000	13,959,000	9,802,000
8	2010 Feb 28	75,769,000	83,765,000	17,102,000	7,997,000	15,293,000	2,408,000	8,583,000	6,044,000	1,829,000	14,438,000	10,071,000
9	2010 Mar 7	76,480,000	84,633,000	17,196,000	8,153,000	15,386,000	2,408,000	8,713,000	6,090,000	1,874,000	14,613,000	10,200,000
10	2010 Mar 14	77,192,000	85,501,000	17,291,000	8,309,000	15,478,000	2,408,000	8,842,000	6,137,000	1,920,000	14,788,000	10,328,000
11	2010 Mar 21	77,903,000	86,369,000	17,386,000	8,465,000	15,570,000	2,408,000	8,971,000	6,184,000	1,965,000	14,963,000	10,456,000
12	2010 Mar 28	78,615,000	87,236,000	17,481,000	8,622,000	15,662,000	2,408,000	9,101,000	6,230,000	2,010,000	15,138,000	10,585,000
13	2010 Apr 4	78,943,000	87,680,000	17,536,000	8,737,000	15,743,000	2,408,000	9,144,000	6,239,000	2,017,000	15,207,000	10,649,000
14	2010 Apr 11	79,272,000	88,124,000	17,592,000	8,852,000	15,824,000	2,408,000	9,188,000	6,247,000	2,024,000	15,276,000	10,713,000
15	2010 Apr 18	79,600,000	88,568,000	17,648,000	8,968,000	15,905,000	2,408,000	9,232,000	6,255,000	2,030,000	15,345,000	10,778,000

*Monthly estimates, by population subgroups, are from the National 2009 H1N1 Flu Survey (NHFS) and the Behavioral Risk Factor Surveillance System survey (4,9,10,14,16,28). Estimates were benchmarked to final season monthly estimates for available age and target groups (9). Adjustments were made to overall population counts and the number of persons vaccinated to account for limitations in either dataset. The distribution of weekly and monthly numbers of persons vaccinated were obtained from the survey data. HR, high risk; NP, not pregnant; HCW, health care worker; Contact, household contacts and caregivers of children <6 mo of age. †Weekly estimates were made by examining overall National 2009 H1N1 Flu Survey weekly vaccination numbers and by using the same distribution to estimate the first 3 weeks of data. For all other weeks, data were estimated by using linear interpolation between point estimates at the start and end of each month. ‡Weeks during which influenza activity was reported during 2009–10 influenza reporting period; 40 –52 indicates last 12 weeks of 2009, and 1–15 indicates first 15 weeks of 2010. §Estimated number of pregnant women from survey data was considered an underestimation because of the definition of pregnant women and low sample size. Therefore, the total number of pregnant women vaccinated during the course of the pandemic was based on data from Moro et al (18). Because the cohort of pregnant women changed during the influenza season, analysis for this group was restricted to NHFS interviews conducted April 4, 2010–Jun 30, 2010. During this time, a new question, “Were you pregnant at any time October 2009 through January 2010?” was added to the NHFS for women 18–64 y to include those pregnant during the major vaccination period. Because no information was available on vaccinations among pregnant women after January, we assumed no further vaccinations were administered, an approach that could have underestimated the effects of A(H1N1)pdm09 virus vaccination on pregnant women.

#### Estimation of Prior Protection of Vaccinated Persons

Our estimates of the number of persons already immune to the A(H1N1)pdm09 virus are based on data for April 12, 2009–April 10, 2010, and an assumed proportion of subclinical cases; we did not include protection from previous years. For our base estimate, we assumed that 30% of all cases were subclinical; this assumption was held constant throughout the pandemic among all subgroups. Data from numerous countries and influenza challenge studies indicate that 24%–36% of the A(H1N1)pdm09 virus cases were subclinical (29–36). We tested the effect of this assumption in our sensitivity analysis. We also assumed that persons who were vaccinated had the same probability of prior infection as the general population.

### Equations

We used the following equations to calculate clinical cases prevented. The equations for prevention of hospitalizations and deaths are identical, except that prior clinical or subclinical infections were not included.

#### Equation 1a

Interim estimated clinical cases prevented by a vaccination program (by population subgroup, at specific points in time, Phase 1) = Doses administered (using estimates from the 2 weeks prior to a specific date) × probability of not having had a prior clinical or subclinical infection (based on original pandemic data) × probability of having a future clinical infection (based on original pandemic data) × vaccine effectiveness

#### Equation 1b

Interim epidemic curve = Original epidemic curve + Estimated cases prevented (Equation 1a)

#### Equation 2a

Estimated cases prevented by a vaccination program (by population subgroup, at specific points in time, Phase 2+) = Doses administered (using the estimates from the 2 weeks prior to a specific date) × probability of not having had a prior clinical or subclinical infection (based on Interim epidemic curve, Equation 1b) × probability of having a future clinical infection (based on Interim epidemic curve, Equation 1b) × vaccine effectiveness

#### Equation 2b

Epidemic curve without a vaccination program = Interim epidemic curve (Equation 1b) + Estimated cases prevented by a vaccination program (Equation 2a)

#### Equation 2c

Final check: Estimated number of clinical cases prevented by a vaccination program (final outcome from final repetition of Equation 2a) = Final Epidemic Curve (final adjustment from Equation 2b) – Original epidemic curve (with a vaccination program)

#### Equation 3

Number needed to treat = number of doses administered/number of medical events (i.e., clinical cases, hospitalizations, or deaths) averted

### Sensitivity Analyses

We conducted sensitivity analyses for 8 scenarios (see below); for each scenario the epidemiologic curve used was identical to that for our base case estimates, assuming that a vaccination program did not exist (Figure 1). For all scenarios except scenario 5, the total number of doses administered each week was the same as the number in our base estimate. We assumed that no children 6 months–9 years of age could have received their second dose until the fifth week of the vaccination program. Therefore, for scenarios 1–4, we assumed that only first doses were administered to children in this age group during the first 4 weeks.

#### Scenario 1: Even Distribution over Time 

To assess the effects of accelerated vaccine uptake among specific groups, we calculated the proportion of total doses administered among each population subgroup over the course of the pandemic. We multiplied the result by the number of doses administered each week; e.g., if a subgroup received a total of 20% of the doses, we assumed that they received 20% each week.

#### Scenario 2: Population Proportions 

We assumed that each population subgroup had a proportionately equal demand for the vaccine. For each subgroup, we set the proportion of vaccine equal to the population proportion (e.g., if a population subgroup represented 10% of the populations, we assumed that the subgroup would be administered 10% of the doses each week).

#### Scenario 3: 2008 Distribution

We used the proportion of doses administered among each subgroup during the 2008 seasonal vaccination campaign. That is, if a population subgroup received 15% of the doses in 2008, we assumed that they used 15% of the doses each week during the 2009 pandemic).

#### Scenario 4: 2009 ACIP Priority Subgroups

To assess the effects of providing the vaccine only to the aforementioned 2009 ACIP priority subgroups, we used the total percentage of doses administered to each group, based on the total 2009 vaccine uptake estimates, but adjusted the denominator of total doses by excluding the non-ACIP priority subgroups. We applied that percentage to the total number of doses administered each week.

#### Scenario 5: Accelerated Start Date

We estimated the effects of moving the start date of the vaccination program to begin 8 weeks to 1 week earlier. We did this by moving the date forward in increments of 1 week.

#### Scenario 6: Vaccine Effectiveness

We examined the outcomes of assuming different vaccine effectiveness. We initially increased vaccine effectiveness to 85% for all health outcomes in population subgroups, except those including persons >65 years of age, for which we increased the effectiveness to 55% for all outcomes. Last, we assumed vaccine effectiveness at 40% for all health outcomes in all population subgroups, except those including persons >65 years of age, for which we assumed 20% effectiveness for all outcomes.

#### Scenario 7: Effectiveness of First Dose for Children

We examined the effects of assuming that, among vaccinated children 6 months–9 years of age, the first dose of vaccine was 20%–40% effective 2 weeks after administration and that vaccine effectiveness reached the levels listed in Table 1 by 2 weeks after the second dose was administered. Some evidence in the published literature shows that 1 dose might have provided some protection (37).

#### Scenario 8: Proportion of Subclinical Cases 

We varied the range of subclinical cases from 0% to 50%. The base estimate was 30%.

## Results

### Health Effects of Vaccination Program

We estimate that during October 3, 2009–April 18, 2010, the A(H1N1)pdm09 virus vaccination program directly prevented 712,908–1,458,930 clinical cases of A(H1N1)pdm09 infection, 3,923–10,393 hospitalizations, and 201–520 deaths (Tables 3,4,5). Based on the number of patients who needed to be treated to prevent 1 additional bad outcome, the vaccination program, as implemented, had the most value for pregnant women and for persons in the ACIP target group who were 25–64 years of age (Tables 3–5).

**Table 3:** Estimated number of cases of influenza prevented by vaccination against influenza A(H1N1)pdm09 virus*

Subgroup	No. clinical cases in absence of vaccination program (range)	No. clinical cases prevented by a vaccination program (range)	No. doses administered to avoid 1 clinical case (range)
6 mo–9 y	12,333,906 (8,766,004–18,088,655)	81,518 (52,081–100,349)	326 (265–511)
10–24 y (10–17 all, 18–24 NP)	13,891,877 (9,879,008–20,374,801)	300,724 (212,953–420,991)	53 (38–75)
Pregnant 18–64 y	1,410,032 (1,004,978–2,062,896)	71,601 (53,084–97,884)	34 (25–45)
25–64 y, HR, NP	8,378,054 (5,957,746–12,286,626)	164,958 (116,575–228,593)	56 (40–79)
25–64 y, HCW, non-HR, NP	3,530,341 (2,510,291–5,178,995)	123,427 (87,287–177,144)	51 (35–72)
25–64, contact with <6 mo, non-HCW, non-HR, NP	1,550,007 (1,101,603–2,276,098)	29,063 (19,904–43,129)	70 (47–102)
25–64 y, noncontact with <6 mo, non-HCW, non-HR, NP	14,734,336 (10,470,235–21,640,930)	163,327 (107,305–248,548)	94 (62–143)
>65 y	6,038,353 (4,290,972–8,868,687)	94,538 (63,719–142,293)	114 (76–169)
Total	61,866,905 (43,980,837–90,777,687)	1,029,157 (712,908–1,458,930)	86 (61–124)
*All values are estimates. NP, not pregnant; HR, high risk; HCW, health care worker; contact, household contacts and caregivers of children <6 mo of age.			

**Table 4:** Estimated number of hospitalizations prevented by vaccination against influenza A(H1N1)pdm09 virus*

Subgroup	No. hospitalizations of persons in groups with no vaccination program (range)	No. hospitalizations prevented by a vaccination program (range)	No. doses administered to avoid 1 hospitalization (range)
6 mo–9 y	54,745 (38,826–80,563)	614 (328–1,090)	43,333 (24,421–81,227)
10–24 y, (10–17 all, 18–24 NP)	63,117 (44,761–92,999)	1,838 (1,179–3,032)	8,654 (5,246–13,489)
Pregnant, 18–64 y	6,481 (4,590–9,582)	446 (298–722)	5,396 (3,336–8,072)
25–64 y, HR, NP	38,060 (26,990–56,074)	1,029 (653–1,707)	8,972 (5,409–14,132)
25–64 y, HCW, non-HR, NP	16,082 (11,394–23,734)	721 (469–1,181)	8,679 (5,294–13,324)
25–64, contact with <6 mo, non-HCW, non-HR, NP	7,020 (4,981–10,338)	163 (104–270)	12,478 (7,528–19,516)
25–64 y, noncontact with <6 mo, non-HCW, non-HR, NP	67,249 (47,743–98,922)	902 (558–1,516)	17,005 (10,124–27,524)
>65 y	27,789 (19,723–40,901)	527 (334–876)	20,444 (12,305–32,278)
Total	280,544 (199,009–413,112)	6,240 (3,923–10,393)	14,193 (8,522–22,575)

*All values are estimates. NP, not pregnant; HR, high risk; HCW, health care worker; contact, household contacts and caregivers of children <6 mo of age.

**Table 5:** Estimated number of deaths prevented by vaccination against influenza A(H1N1)pdm09 virus*

Subgroup	No. deaths without a vaccination program (range)	No. deaths prevented due to a vaccination program (range)	No. doses administered to avoid 1 death (range)
6 mo–9 y	759 (538–1,117)	9 (5–15)	3,087,138 (1,745,154–5,761,939)
10–24 y, (10–17 all, 18–24 NP)	1,028 (729–1,514)	30 (20–50)	525,012 (319,229–814,797)
Pregnant 18–64 y	533 (378–789)	37 (25–60)	64,787 (40,177–96,492)
25–64 y, HR, NP	3,077 (2,182–4,533)	84 (54–139)	109,638 (66,300–171,951)
25–64 y, HCW, non-HR, NP	1,175 (833–1,735)	53 (35–87)	117,312 (71,786–179,325)
25–64, contact with <6 mo, non-HCW, non-HR, NP	766 (544–1,128)	18 (12–30)	112,945 (68,351–175,889)
25–64 y, noncontact with <6 mo, non-HCW, non-HR, NP	3,792 (2,692–5,578)	52 (32–86)	297,838 (177,870–479,999)
>65 y	1,653 (1,173–2,433)	32 (20–53)	339,494 (204,961–533,711)
Total	12,783 (9,069–18,826)	315 (201–520)	281,305 (170,343–439,832)

* All values are estimates. Vaccinations beginning at week 40 with a distribution of the vaccines as outlined in Table 2. NP, not pregnant; HR, high risk; HCW, health care worker; contact, household contacts and caregivers of children <6 mo of age.

### Effects of Targeting Subgroups

The estimated numbers of clinical cases prevented under different (assumed) prioritization strategies are shown in Table 6. In the 4 sensitivity scenarios related to prioritization strategies, the ranges of estimated total cases prevented overlap substantially. However, the effect on each population subgroup varies considerably. For example, if we focus solely on children <9 years, we estimate that during the 2009 pandemic ≈81,518 (range 52,081–100,349) A(H1N1)pdm09 infections were prevented among this population subgroup. However, by entering the same number of doses and same effectiveness, but adjusting the timing of administration by group (Scenario 1), we calculated that the number of cases prevented in this population subgroup would increase to ≈131,000 ( range 91,000–164,000). In Scenario 2, in which we assumed children 6 months–9 years of age received 9% of all vaccines administered (i.e., population proportional), cases prevented decreased to ≈58,000 (range 40,000–72,000). If no changes had been made to the ACIP recommendations and the rate of vaccine uptake among the different population subgroups had been similar to uptake of the 2008 seasonal influenza vaccine (17,38) (Scenario 3), we would expect the number of cases prevented among children <9 years of age to be ≈65,000 (range 45,000–82,000). This would have been ≈80% of what was estimated during the A(H1N1)pdm09 pandemic. This projected decrease in cases averted indicates that this population subgroup would not have benefitted from such a change in policy. Last, if the A(H1N1)pdm09 virus vaccine had been administered exclusively to those in the ACIP priority groups, we estimate that the number of cases that would have been prevented among children aged <9 years would be ≈186,000 (range 129,000–233,000); under this assumption, 43% of this ACIP target group would be fully vaccinated, compared with an estimated 27% that actually were vaccinated.

**Table 6:** Sensitivity analysis showing number of clinical cases prevented by vaccination against influenza A(H1N1)pdm09 virus for different scenarios of vaccine distribution*

Subgroup	Base case estimate (range)†	Scenario			
		1: even distribution over time (range)‡	2: distribution based on population proportion (range)§	3: 2008 distribution (range)¶	4: ACIP priority subgroups (range)#
6 mo–9 y	81,518 (52,081–100,349)	131,170 (90,932–164,352)	57,511 (39,869–72,060)	65,093 (45,125–81,559)	186,041 (128,970–233,103)
10–24 y (10–17 all, 18–24 NP)	300,724 (212,953–420,991)	279,715 (196,606–392,577)	310,656 (218,355–436,003)	249,981 (175,708–350,847)	396,725 (278,851–556,801)
Pregnant, 18–64 y	71,601 (53,084–97,884)	44,486 (31,726–60,936)	30,506 (21,756–41,787)	14,809 (10,561–20,285)	63,096 (44,998–86,427)
HR, 25–64 y	164,958 (116,575–228,593)	168,521 (119,243–233,197)	183,417 (129,784–253,810)	73,157 (51,765–101,234)	239,017 (169,125–330,749)
HCW, 25–64 y	123,427 (87,287–177,144)	100,229 (69,407–144,610)	82,764 (57,313–119,413)	41,099 (28,460–59,297)	142,157 (98,441–205,104)
Contact with <6 mo	29,063 (19,904–43,129)	28,861 (19,686–42,794)	37,583 (25,634–55,726)	151,525 (103,351–224,675)	40,935 (27,920–60,696)
25–64 y (all others)	163,327 (107,305–248,548)	197,372 (133,316–297,625)	366,354 (247,455–552,439)	278,226 (187,928–419,547)	0
>65 y	94,538 (63,719–142,293)	99,116 (67,121–148,741)	103,402 (70,023–155,172)	197,547 (133,778–296,454)	0
Total	1,029,157 (712,908–1,458,930)	1,049,470 (728,037–1,484,834)	1,172,194 (810,188–1,686,411)	1,071,437 (736,676–1,553,899)	1,067,971 (748,306–1,472,881)
	Assumed % distribution by week**				
6 mo–9 y, 1st dose††	20	20	9	10	28
6 mo–9 y, 2nd dose	10	10	4	5	14
10–24 y (10–17 all, 18–24 NP)	18	18	20	16	25
Pregnant, 18–64 y	3	3	2	1	4
HR, 25–64 y	10	10	11	5	15
HCW, 25–64 y	7	7	6	3	10
Contact with <6 mo	2	2	3	12	3
25–64 y (all others)	17	17	32	24	0
>65 y	12	12	13	24	0

*Data reflect calculations made in scenarios 1 –4. ACIP, Advisory Committee on Immunization Practices; NP, not pregnant; HR, high risk; HCW, health care worker; contact, household contacts and caregivers of children <6 mo of age. †Total number of doses administered to each population subgroup (Table 2). ‡For each population subgroup, this scenario assumes that the group received the same proportion of the total number of doses; the proportions were applied to the total number of doses administered each week (Table 2, Appendix). § It was assumed that the distribution of vaccines was proportional to the population. ¶Distribution of vaccine was based on estimates of estimated 2008 seasonal vaccine uptake (17,38). #Distribution of vaccine was based exclusively on ACIP priority groupings. The proportion of doses administered was based on the proportion of doses administered to persons in each of the subgroups during the A(H1N1)pdm09 virus vaccination program, while excluding the non ACIP subgroups. **In scenarios 1–4, the epidemiologic curve was based on the estimated A(H1N1)pdm09 vaccination curve, for which no vaccination program was assumed (Figure 1). We also assumed that the total number of vaccines administered each week remained exactly the same as outlined in Table 2, Appendix. ††For scenarios 1–4, we assumed that the 6 mo– 9 y age group required 2 doses and that a 4 wk delay was required between the first and second dose. We also assumed that no children 6 mo–9 years of age could have received their second dose until the fifth week of the vaccination program. Therefore, any doses during the first 4 wk that would have been proportioned as a second dose were added as a first dose.

### Effects of Timing of Vaccination Administration

**Figure 2:**
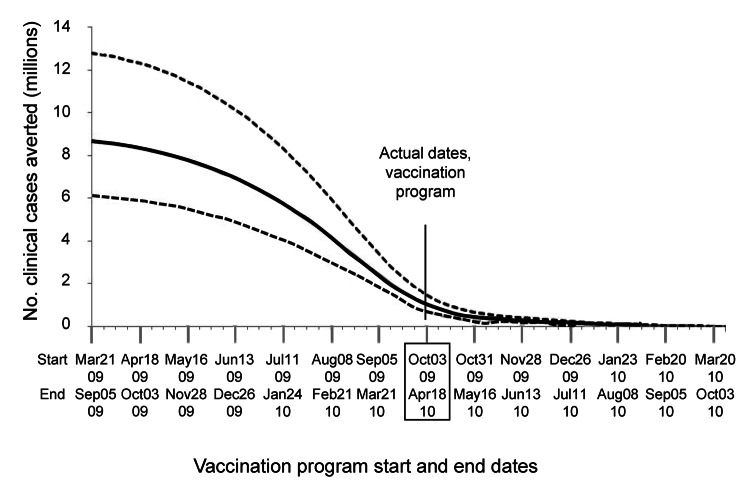
Comparison of the effects of shifting hypothetical start and end dates on the number of clinical cases prevented by the influenza A(H1N1)pdm09 virus vaccination program in the United States. Doses administered by week and program duration were unchanged from actual program (Table 2). Solid line represents the best estimate; dotted lines represent ranges. October 3, 2009–April 18, 2010, is actual vaccination program period; all other periods are hypothetical. See Table 7 for additional data.

The effects of earlier vaccine administration on the number of clinical cases prevented are presented in Table 7 and Figure 2. If the entire A(H1N1)pdm09 virus vaccine program had begun 1 week earlier, the number of clinical cases prevented would have increased by ≈27% more than the base estimate. If it had begun 2 weeks earlier than the actual date, the number of cases prevented would have been ≈59% greater than the base estimate; moving the program ahead by 8 weeks would have resulted in a ≈306% increase in cases prevented compared with the base estimate.

**Table 7:** Sensitivity analyses showing estimates of clinical cases prevented by acceleration of vaccination against influenza A(H1N1)pdm09 virus*

Dates of vaccination program	Point estimate	Range
Hypothetical dates		
2009 Aug 08–2010 21 Feb	4,176,031	2,974,975–5,970,682
2009 Aug 15–2010 28 Feb	3,742,600	2,674,232–5,322,588
2009 Aug 22–2010 07 Mar	3,299,591	2,366,468–4,668,558
2009 Aug 29–2010 14 Mar	2,855,894	2,054,754–4,020,843
2009 Sep 05–2010 21 Mar	2,422,481	1,747,781–3,398,603
2009 Sep 12–2010 28 Mar	2,010,198	1,450,291–2,817,245
2009 Sep 19–2010 04 Apr	1,633,200	1,171,673–2,292,018
2009 Sep 26–2010 11 Apr	1,303,621	922,931–1,836,514
Actual dates		
2009 Oct 03–2010 18 Apr†	1,029,157	712,908–1,458,930

*The epidemic curve that was used to generate these estimates was the base case estimate, which was based on the assumption that a vaccination program did not exist. Data reflect calculations made for scenario 5 by estimating effects of moving the start date of the program to begin 8 weeks to 1 week earlier. †See Table 2, Appendix, wwwnc.cdc.gov/EID/article/19/3/12-0394-T2.htm.

### Outcomes of Vaccine Effectiveness

The vaccine administered during the 2009–2010 A(H1N1)pdm09 vaccine program was 62%% effective, and was calculated to have prevented ≈1,000,000 (range 712,908–1,458,930) clinical cases. If the vaccine had been more effective (85% effective for all groups, except for persons >65 years of age, for whom effectiveness was assumed to be 55%), 983,671–2,004,053 clinical cases would have been prevented (≈38% more than in the base estimate). If the vaccine had been less effective (40% effective for all groups, except for persons >65 years of age, for whom it was assumed to be 20% effective), 442,971– 907,688 clinical cases would have been prevented (≈38% fewer than in the base estimate) (Table 8).

**Table 8:** Results of sensitivity analyses to estimate number of cases, hospitalizations, and deaths prevented by vaccination against influenza A(H1N1)pdm09 virus obtained with various vaccine effectiveness scenarios*

Outcomes prevented	Base estimate of vaccine effectiveness (range)†	Lower vaccine effectiveness (range)‡	Higher vaccine effectiveness (range)§
Clinical cases	1,029,157 (712,908–1,458,930)	639,449 (442,971–907,688)	1,418,678 (983,671–2,004,053)
Hospitalizations	6,240 (3,923–10,393)	3,857 (3,923–6,418)	8,674 (3,923–14,461)
Deaths	315 (201–520)	193 (124–319)	438 (279–723)

*Data reflect calculations made for scenario 6, outcomes of assuming different vaccine effectiveness. †Assumed 62% effectiveness for all groups except those >65 y, for whom 43% effectiveness was assumed. ‡Assumed 40% effectiveness for all groups except the elderly, for whom 20% effectiveness was assumed.  §Assumed 85% effectiveness for all groups except the elderly, for whom 55% effectiveness was assumed.

### Effects of the 2-dose Vaccine Program for Children

In our base case estimate, we assumed 0% effectiveness for a single dose of vaccine and 63% effectiveness for a second dose administered 4 weeks later for children <9 years, and we estimated that vaccination prevented 52,081–100,349 clinical cases among persons in this age group (Table 9). Assuming that an initial dose was 20% effective, 152,420–268,852 clinical cases would have been prevented, and assuming an initial dose was 40% effective, 256,510–439,714 clinical cases would have been prevented. This striking difference between the base estimate and the other estimates occurred primarily because only ≈51% of the children who received their first dose also received a second dose, and children who received only 1 dose were not considered protected in the base case estimate.

**Table 9:** Results of sensitivity analyses to determine the impact of the effectiveness of the first dose of vaccine against influenza A(H1N1)pdm09 virus among children 6 months–9 years of age*

Outcome prevented	Base estimate: 62% vaccine effectiveness 2 wk after dose 2†	Sensitivity estimate (range)	
		20% Vaccine effectiveness 2 wk after dose 1 and 62% effectiveness 2 wk after dose 2‡	40% Vaccine effectiveness 2 wk after dose 1 and 62% effectiveness 2 wk after dose 2§
Clinical cases	81,518 (52,081–100,349)	212,363 (152,420–268,852)	347,323 (256,510–439,714)
Hospitalizations	614 (328–1,090)	1,473 (906–2,294)	2,393 (1,520–3,964)
Deaths	9 (5–15)	21 (13–35)	33 (21–55)

*Data reflect calculations made for scenario 7 by estimating changes in assumed effectiveness first dose of vaccine among children 6 months–9 years of age. †1 dose achieves 0% effectiveness against clinical cases, hospitalizations, and deaths; 2nd dose 4 wk later is 62% effective against hospitalizations and deaths 2 weeks after administration. ‡1 dose achieves 20% effectiveness against clinical cases, hospitalizations, and deaths after 2 wk; 2nd dose 4 wk later achieves 62% against hospitalizations and deaths 2 weeks after administration.  §1 dose achieves 40% effectiveness against clinical cases, hospitalizations, and deaths after 2 wk; 2nd dose 4 wk later achieves 62% against hospitalizations and deaths 2 weeks after administration.

### Effects of Subclinical Cases

In our base estimate we assumed that 30% of all cases were subclinical. When we assumed that 50% of all cases were subclinical, the estimated number of clinical cases prevented was 87% of the base estimate. When we assumed that 0% of all cases were subclinical, the number of clinical cases prevented was 110% of the base estimate (Table 10).

**Table 10:** Results of sensitivity analyses to determine effects of assumed percentages of subclinical cases of influenza A(H1N1)pdm09 virus infection*

Assumed proportion subclinical cases	No. clinical cases prevented (range)
50%	891,682 (651,567–1,135,546)
30% (base estimate)	1,029,157 (712,908–1,458,930)
0%	1,133,734 (759,341–1,706,714)

*Data reflect calculations made for scenario 8 by changing assumed percentages of subclinical cases of influenza.

## Discussion 

We estimated that ≈1 million clinical cases, 6,000 hospitalizations, and 300 deaths were prevented among persons who received the monovalent A(H1N1)pdm09 virus vaccine. Approximately 60% of clinical cases prevented were among persons 6 months–24 years of age and among those 25–64 years, including pregnant women, who were considered at high risk for influenza-related complications. We found that the effects of the vaccination program were greatly influenced by the timing of vaccine administration and by vaccine effectiveness.

Vaccine prioritization recommendations were made in July 2009 based on limited epidemiologic data, previous experience with immunologic responses to novel vaccine antigens, projections about when and how much vaccine would be initially available, and previous public engagement and expert opinion summaries about public values and preservation of societal functions (5,6). These factors led to a policy that identified and focused on children, pregnant women, and medical personnel as population subgroups who should receive vaccine as early in the program as possible. Uncertainty in the epidemiologic data makes it difficult to accurately determine exactly how many cases, hospitalizations, or deaths would have been prevented under any given scenario. However, the results of our sensitivity analyses indicate that the effects of the 2009 ACIP recommendations were similar, and for some subgroups even better, than those for other vaccine prioritization strategies.

This study has several limitations. We did not directly account for the effects of any other interventions (e.g., antiviral drugs, school closures, facemasks, improved management of clinical cases); we assumed these to remain constant, with or without a vaccination program. We did not estimate the curve beyond April 10, 2010, which may have resulted in a slight underestimation of the effects. However, influenza-like illness data for the United States indicated that it was unlikely that many cases occurred after April 2010 (www.cdc.gov/h1n1flu/updates/us/051410.htm [cited 2013 Jan 11]). We did not directly account for any vaccine-induced herd immunity. Estimates of A(H1N1)pdm09 virus vaccination coverage were based on survey data and subject to bias from low sample sizes from specific population subgroups and misclassification of vaccination status. Weekly vaccination estimates were interpolated. One of our sensitivity analyses illustrated the importance of the assumed level of vaccine effectiveness (Tables 8, 9). The delays we assumed between vaccination and effective protection could also have affected the estimates.

This study highlights the benefits of earlier, proactive (as opposed to reactive) vaccination programs. However, current influenza vaccine production technology is limited in how quickly large-scale vaccine production can be achieved, and the public health community cannot accurately predict the arrival of a pandemic. This study also demonstrates that the 2009 prioritization of specific subgroups in vaccine administration was not inferior to other vaccination strategies. In addition, this study highlights the need for better data on the effectiveness of influenza vaccine. Influenza vaccine effectiveness estimates vary considerably according to season, yet clearly they can greatly affect the overall results and conclusions of programs for policy makers.

## Conclusions 

Future influenza pandemics are likely to differ in several ways, including in severity (patients’ signs and symptoms were mild during the 2009 pandemic), basic reproductive rate of the virus, virus subtype, subgroups affected, public acceptance of vaccination, vaccine safety profile, and vaccine effectiveness. The major factor influencing the effects of the 2009 subtype H1N1 vaccination program was that the amount of vaccine available early in the epidemic (when the effects of vaccination would be greatest) was limited. Thus, a major priority is to invest in research that can reduce production time (e.g., developing prepandemic vaccines [38] and new types of vaccines and production technologies) and the quantity of vaccine initially available (e.g., through antigen-sparing strategies and adjuvants). Robust immunization programs that can more efficiently provide vaccines to targeted groups, faster production of larger supplies of vaccine, and consistent messaging that engenders public confidence in vaccine programs and demand for vaccination (e.g., messaging from public health officials; the media; and community groups, such as churches, daycare facilities, and schools) are factors that must be addressed in preparing for national outbreaks and pandemics.

Technical Appendix Spreadsheet-based model to calculate the impact of a vaccination program against influenza A(H1N1)pdm09 virus in the United States. Model allows the user to adjust many of the input variables.
